# Implementation of a Traceback Testing Program for Ovarian Cancer: Findings from the FACTS Study

**DOI:** 10.3390/cancers17071154

**Published:** 2025-03-29

**Authors:** Nora B. Henrikson, M. Cabell Jonas, Paula R. Blasi, Adam H. Buchanan, Pim Suwannarat, Kathleen Leppig, Aaron Scrol, Tracey Leitzel, Adrienne N. Deneal, Daniela Canedo, Arvind Ramaprasan, Sundeep S. Basra, Jennifer Brown, Marilyn Odums, Yirui Hu, Katrina M. Romagnoli, Estella Khieu, Elsa Balton, Saumya Patel, Muki Kunnmann, Dina Hassen, Jing Hao, Meredith Lewis, Rachel Schwiter, Jessica Goehringer, Heather M. Ramey, Shanshan Gustafson, Katrina Hsieh, Ilene Ladd, Alanna K. Rahm

**Affiliations:** 1 Kaiser Permanente Washington Health Research Institute, 1730 Minor Ave, Suite 1360, Seattle, WA 98101, USA; 2 Mid-Atlantic Permanente Research Institute, 700-B 2nd Street NE, Washington, DC 20002, USA; 3 Geisinger Department of Genomic Health, 658 Center Street, Danville, PA 17822, USA; 4 Division of Medical Genetics, Department of Medicine, University of Washington, 1705 NE Pacific St., Seattle, WA 98195, USA; 5 Swedish Health Services Maternal and Fetal Specialty Center, Seattle, WA 98104, USA

**Keywords:** ovarian cancer, cancer prevention, gynecologic cancer risk reduction

## Abstract

**Background:** Traceback testing—identifying and offering testing to people with previous cancer diagnoses who have not received current standard genetic testing—could benefit patients and their at-risk relatives. Methods: We conducted a multisite, nonrandomized pilot implementation study of a Traceback program at three integrated United States health systems. We assessed the reach, fidelity, effectiveness, and acceptability of the program using quantitative and qualitative methods. Results: We identified 597 eligible individuals using administrative data and manual chart review. We attempted to reach everyone identified (100% fidelity). We successfully contacted 354 people, for a reach of 59% of confirmed eligible individuals. In total, 133 people completed Traceback genetic testing. Ten of these (8%) received pathogenic or likely pathogenic results;. Nine of these ten people received positive results for which cascade testing of at-risk relatives would be indicated. None of their relatives underwent cascade testing during the study period. Thirty-six received variants of uncertain significance (VUS). Traceback programs were acceptable to participants and implementers and thought to be applicable to other genetic screening conditions. The time and resources required to accurately identify Traceback-eligible individuals are likely determinants of future sustainability. Conclusions: Education about free cascade testing, reminder calls to probands, and offers to directly contact at-risk relatives did not result in cascade testing in this pilot study. However, participant and implementer discussions suggest that the potential benefits of Traceback programs and high participant acceptability are worthy of further study.

## Simple Summary

Genetic testing can help people with cancer and their families. Traceback testing finds people with cancer who missed genetic testing at diagnosis and offers it to them. We studied a Traceback program in three U.S. health systems. We identified 597 eligible patients and reached 59% of them. Of those reached, 133 (38%) completed genetic testing. Ten people (8% of those tested) had results that indicated increased cancer risks. However, no at-risk relatives completed free testing, despite reminders and support. Participants and staff thought that the program was important, but the extra work to accurately identify people who are eligible for Traceback testing could limit long-term success. Overall, the program found and reached people who were eligible for Traceback testing, but no at-risk relatives received testing during the study. Future studies can explore the program’s potential.

## 1. Introduction

Ovarian cancer is the sixth leading cause of cancer death in women. When detected early, the relative survival rate of ovarian cancer is over 90% [1], presenting an opportunity for primary prevention and early detection that could reduce the human suffering associated with ovarian cancer. Pathogenic variants in *BRCA1* and *BRCA2* and several other genes are risk factors for ovarian cancer [2], conferring a 17–44% lifetime risk of ovarian cancer for females with pathogenic variants in the *BRCA1* or *BRCA2* genes, as well as increased risks for breast (72%) and other cancers [3]. Current guidelines recommend genetic testing of all individuals with newly diagnosed ovarian cancer [4]. However, genetic testing at diagnosis has been limited, leaving many families without knowledge of potential pathogenic variants in the family. Furthermore, guidelines have evolved over time, leaving many people without current standard genetic testing. At-risk relatives of patients with a known pathogenic variant are recommended to be referred for familial variant testing, known as cascade testing. Identified carriers can choose surveillance for early cancer detection and/or risk-reducing surgery, both of which are associated with substantially improved health outcomes [5]. However, cascade testing uptake among eligible relatives is low—only about 20–30% [6]—even when there is no charge billed to family members for cascade testing [7]. This represents a missed opportunity for implementation of precision medicine and improved health outcomes in families.

The National Cancer Institute’s (NCI) Traceback framework for family-based outreach consists of three phases: (1) identification of individuals with ovarian cancer without prior genetic testing (i.e., probands); (2) genetic testing of these individuals; and (3) cascade testing of at-risk family members [6,8]. Following this, the Feasibility and Acceptability of Cascade Traceback Screening (FACTS) study implemented Traceback testing for living ovarian cancer survivors without documentation of current standard genetic testing at diagnosis in three United States health systems [9]. Here, we describe the results of our implementation and mixed methods evaluation.

## 2. Materials and Methods

We conducted a multisite, nonrandomized pilot implementation study. Our project was informed by the Conceptual Model for Implementation Research, which provides guidance for measuring outcomes across multiple levels [10]. The model also both distinguishes between and links implementation processes and outcomes and informed data collection and interpretation of results across all aims. Study activities were approved by the Institutional Review Board of Kaiser Permanente Interregional IRB and by Geisinger IRB.

We implemented and evaluated the Traceback program at three integrated health systems: Kaiser Permanente Washington (KPWA), Kaiser Permanente Mid-Atlantic States (KPMAS), and Geisinger (GE). These three systems represent geographic diversity, racial and ethnic diversity, diversity of insurance models [11], and the opportunity to offer genetic testing to individuals and their at-risk relatives.

### 2.1. Population and Eligibility

Inclusion criteria included living and currently receiving care in one of the three health systems with a prior diagnosis of ovarian, peritoneal, or fallopian tube cancer and no history of current standard panel germline genetic testing for hereditary cancer risk at diagnosis. Patients diagnosed since 1980 and still living were eligible, as were individuals who were tested and received a negative result prior to 2014, when current panel testing became standard. Exclusion criteria included being in hospice care, having an ovarian tumor with borderline histology, and inability to provide verbal consent.

### 2.2. Intervention

The Traceback framework includes three phases to facilitate the identification of and genetic testing for inherited pathogenic variants: (1) identifying eligible probands, (2) proband testing, and (3) cascade testing of at-risk relatives [6]. Each of the three study sites implemented these three phases. First, we identified potentially eligible participants using administrative and tumor registry data. All sites used the same programming specifications to identify eligible participants in their tumor registries. KPMAS also used diagnosis codes to identify eligible patients within two additional internal patient databases. Unexpectedly, manual chart review by a clinician was necessary to confirm eligibility, in particular prior diagnosis (e.g., for tumors with borderline histology) and presence or absence of previous germline genetic testing that was not reliably accessible within the administrative or tumor registry databases (e.g., in scanned PDF reports). Therefore, we conducted manual chart reviews to confirm eligibility before contacting individuals.

To implement testing of eligible probands, we designed intervention materials and workflows based on our preliminary work [12]. To design the Traceback programs, we conducted interactive qualitative interviews using human-centered design methods with patients at each site (total *n* = 70) with ovarian, fallopian tube, or peritoneal cancer (probands) and people with a family history of ovarian cancer (relatives). Participants preferred to be approached using personalized communication, with access to a physician discussion if needed for follow-up questions. They preferred messaging focused on clinical benefits to relatives, rationale for testing, and ease of access to testing [12].

**Figure 1:**
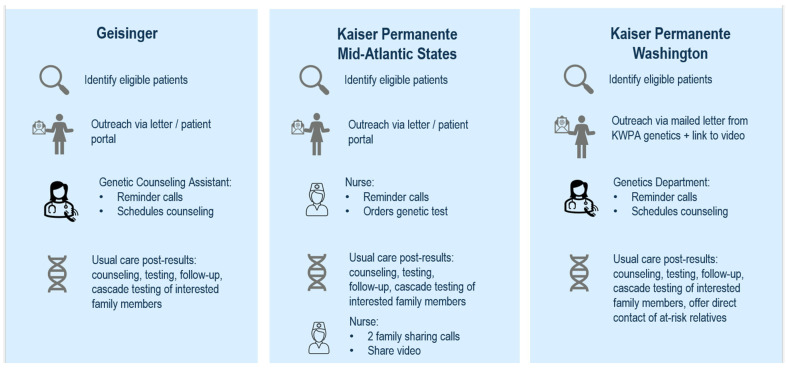
Overview of Traceback testing programs at each site.

Each site designed their specific workflows according to local context and workflow needs and adapted the same recruitment materials. Descriptions of each site’s intervention processes are shown in Figure 1. Invitations to participate in Traceback testing were sent via mail or patient portal, with follow-up reminder calls to schedule a pre-test genetic counseling visit for interested participants (KPWA and GE), with subsequent laboratory-based sample collection. At KPMAS, the language in outreach materials differed slightly based on whether the patient had out-of-date testing (prior to 2014) or no evidence of testing. At KPMAS, in lieu of pre-test genetic counseling, a study team nurse conducted outreach reminder calls and ordered a genetic testing kit to be sent directly to interested participants’ homes. All participants received testing via the Invitae Common Hereditary Cancer Panel (San Francisco, CA, USA), a primary panel of 48 genes (*APC*, *ATM*, *AXIN2*, *BAP1*, *BARD1*, *BMPR1A*, *BRCA1*, *BRCA2*, *BRIP1*, *CDH1*, *CDK4*, *CDKN2A*, *CHEK2*, *CTNNA1*, *DICER1*, *EPCAM*, *FH*, *GREM1*, *HOXB13*, *KIT*, *MBD4*, *MEN1*, *MLH1*, *MSH2*, *MSH3*, *MSH6*, *MUTYH*, *NF1*, *NTHL1*, *PALB2*, *PDGFRA*, *PMS2*, *POLD1*, *POLE*, *PTEN*, *RAD51C*, *RAD51D*, *SDHA*, *SDHB*, *SDHC*, *SDHD*, *SMAD4*, *SMARCA4*, *STK11*, *TP53*, *TSC1*, *TSC2*, *VHL*).

All 3 sites used their usual care procedures for return of results and clinical follow-up. Individuals with positive results were recommended to inform their at-risk relatives of their result and to inform relatives of the availability of free cascade testing from the testing laboratory. Individuals with VUS were informed of the result, that their family members should not have genetic testing for the VUS as the presence or absence is not sufficient to inform risk, and that variant classification may be updated in the future as additional knowledge is gained. At KPMAS, after the results were returned, the nurse additionally offered reminder calls and shared a video made by the study team describing Traceback testing for participants to share with their at-risk relatives. At KPWA, the genetic counselor offered to directly contact at-risk relatives to recommend cascade testing, consistent with ethical guidance that suggests contacting relatives directly is acceptable with the proband’s consent [13,14]. As part of other ongoing clinical efforts, Geisinger was also able to offer direct contact to at-risk relatives with proband consent later in the program implementation. Enrollment was completed between March 2022 and March 2023. Results were returned between August 2022 and July 2023.

### 2.3. Data Collection and Analysis

To assess reach, fidelity, and effectiveness, all sites monitored and recorded the numbers of patients who were eligible, invited, and tested. We assessed testing uptake and results of proband genetic testing and cascade testing in relatives across the three sites. To assess representativeness, we also collected demographic information, including age at diagnosis and at the time of our eligibility assessment. Information regarding stage at diagnosis was not reliably available, so was not included. We collected the numbers of pathogenic results and variant of uncertain significance (VUS) results returned to patients. To evaluate cascade testing uptake, we recorded the number of first or second degree (if FDR was deceased) at-risk relatives for each participating proband. We worked with Invitae to receive the numbers of at-risk relatives completing cascade testing during Invitae’s free 150-day testing period offered to at-risk relatives.

To assess acceptability, we conducted qualitative interviews with program participants. We invited all individuals invited to Traceback testing to participate in an interview, regardless of whether they accepted genetic testing or not. About four months after participants were invited to receive genetic testing, we contacted participants again via letter, secure message, or phone call to invite them to participate in a telephone interview about their experience with the Traceback program. We followed a purposeful consecutive sampling approach by scheduling interviews with all those interested until we reached our goal of interviewing at least 45 probands, or about 15 per study site. Participants received USD 25 honoraria for their time. All interviews were conducted between August 2022 and July 2023.

Trained interviewers (P.R.B., A.D., C.J., D.C., J.G., T.L., A.M.K., S.P., R.S.) conducted one-on-one interviews with consenting participants; in some instances, a second interviewer joined the call to assist with taking notes. Interviewers used a secure Microsoft Teams conference line to conduct and record the audio-only interviews, which lasted 20–60 min. Interviewers followed a semi-structured interview protocol. Questions related to the reasons for participating, reaction to and experiences with the Traceback program, impacts of the program, and suggestions for improvement (the interview guide is available in the Supplementary Materials). Interviewers wrote brief field notes following each interview. Audio recordings were transcribed verbatim using professional transcription services. All transcripts, field notes, and memos identified participants by study identification number only.

We used Atlas.ti (Berlin, Germany; KPWA and Geisinger) and NVivo (Burlington, MA; KPMAS) to store, organize, and code anonymized interview transcripts. We conducted thematic analysis using a template analysis approach that relied on both a priori and emergent codes [15,16,17]. We developed a priori codes based on our research questions and interview field notes and we identified additional codes from the data. After piloting and finalizing our coding approach and definitions, we independently coded the remaining transcripts and drafted coding memos describing preliminary themes and supporting quotations. We examined similarities and differences in findings across study sites. The study team engaged in iterative discussions to further identify connections in the data, interpret findings, and focus the scope of the analysis. Investigators were not known to participants prior to this study. The authors practiced reflexivity by documenting and discussing the potential influence of their personal characteristics and perspectives on their interviews and analyses.

To assess stakeholder perspectives on Traceback programs, we conducted virtual, semi-structured discussions with program implementers at each FACTS clinical site between July and September 2023. Implementers included medical geneticists, genetic counselors, genetic counseling assistants, and nurses who were involved in the Traceback program at each clinical site. Discussions took place after the Traceback program patient outreach and genetic testing had concluded at each site. The study investigators invited implementers via phone or email. Discussion questions were guided by the Conceptual Model for Implementation Research and focused on implementation, service, and client outcomes. Topics included overall experience with the program, perceived benefits of the program, factors influencing sustainability, and improvements needed. The Acceptability of Intervention Measure (AIM) and Intervention Appropriateness Measures (IAM [18]) were administered at the close of each interview, with response options of Agree or Disagree (adapted from original 5-point scale).

Discussions were led and attended by study investigators (A.K.R., C.J., N.B.H.). Discussions were audio-recorded on a secure Microsoft Teams conference line. Investigators took notes on interview findings from the recordings, and prepared written summaries of each site’s interviews. Through a series of analysis meetings, the team summarized the findings across discussions.

## 3. Results

Across the three sites, we identified 6386 people through administrative data who were potentially eligible for Traceback testing. Of these, 597 were confirmed eligible after manual chart review. Exclusions included individuals with tumors not known to be associated with hereditary cancer predisposition, individuals with a non-ovarian primary cancer that had spread to the ovary, and those with benign ovarian tumors. Individuals with serous or endometrioid ovarian carcinomas were included on a case-by-case basis during manual chart review by clinicians at KPMAS and KPWA, and included at Geisinger. We attempted to reach everyone identified (100% fidelity). Of these, 173 (29%) passively refused, defined as no response to outreach. We successfully contacted 354 people, for a reach of 59% of confirmed eligible individuals (range 48–68%).

In total, 133 people (22% of those eligible, 38% of reached individuals) completed Traceback genetic testing (Table 1). Participants were representative of non-participants for age at diagnosis, race/ethnicity, and ovarian vs. other cancer type (fallopian or peritoneal). Age at eligibility assessment was different at two of the three sites. At KPMAS, participants were older at eligibility assessment than nonparticipants (68.0 vs. 65.2, *p* = 0.024), as were KPWA (73.8 vs. 70.7, *p* = 0.042). Age at diagnosis was similar between sites (mean age: 57.4 years). Between sites, KPWA had significantly older participants at enrollment/eligibility assessment compared to KPMAS and Geisinger (73.8 years vs. 68.0 and 69.8; *p* = 0.029 for differences across sites). KPMAS had the most diverse participants, with 35% reporting Black and 12% Asian race, compared to 5% Black and 5% Asian at KPWA and 0% Black and 6% Asian at Geisinger (*p* < 0.001), reflecting their patient population and diversity-focused sampling strategy. Most (92%) participants had ovarian cancer; 8% had peritoneal or fallopian cancer. Rates of completing testing varied across sites, from 8% to 33%.

Table 2 shows the genetic testing and cascade testing results. In total, 10 Traceback testing participants received pathogenic or likely pathogenic results, representing a positive rate of 8% of people completing testing and 3% of all confirmed eligible for Traceback testing, including seven with a pathogenic variant in a gene with strong or definitive evidence of association with ovarian cancer (*ATM* [2], *BRCA1* [1], *BRCA2* [2], *RAD51D* [2]; range of pathogenic findings across sites was 5–9% of people completing testing; Table 3). Thirty-six participants received variants of uncertain significance per each site’s normal clinical practice; VUS rates were similar across sites. Nine of ten people received positive results for which cascade testing of at-risk relatives would be indicated. Genetic counselors identified 58 eligible at-risk relatives among these 9 participants. No probands provided consent for the study team to contact their relatives directly. Over the study period, no at-risk relatives underwent cascade variant testing, which was provided at no charge by the testing laboratory.

### 3.1. Traceback Participant Experience

We completed interviews with 47 probands, including 18 from Geisinger, 16 from KPWA, and 13 from KPMAS (Table 4). All participants reported female gender, and their median age was 69. Most participants (76.6%) were of white race, and none reported Hispanic ethnicity. More than half of the participants (57.4%) reported having a 4-year college degree or more education, and a quarter (25.5%) reported an annual household income of more than USD 75,000. Demographic characteristics were similar across study sites, though KPMAS had a larger proportion of Black participants (38.5%) compared with all sites combined (12.8%), and Geisinger had a smaller proportion of participants reporting incomes greater than USD 75,000 reflecting its mostly rural population . Though we looked for differences and similarities across the three study sites, we did not detect differences by site in how participants described their experiences of the Traceback program; therefore, the following results include data across all sites.

Participants generally had positive reactions to the Traceback program’s invitation to have genetic testing, noting they trusted their health system and appreciated the opportunity to learn more about their genetic risk (Table 5). Only three participants reported surprise, confusion, or a negative reaction to the invitation, noting they did not understand why they were receiving the invitation many years after their initial cancer diagnosis.

Thirty of the forty-seven interview participants (63.8%) had accepted the invitation to receive genetic testing. Of the 17 participants who initially declined genetic testing, 5 changed their mind during the interview and agreed to receive testing. Reasons for accepting genetic testing included learning information that could affect participants’ health and the health of family members, as well as an interest in contributing to research and helping future patients. Reasons for declining testing included the cost of testing, logistical barriers such as scheduling challenges, and a belief that the information would not be useful to them or their family (e.g., because the participant is elderly and/or already aware of an increased cancer risk).

Participants who received genetic counseling and testing generally reported positive experiences with the process, noting the sample collection process was easy, straightforward, and often could be completed as part of an unrelated clinic visit. The few instances of negative experiences were related to miscommunications or mix-ups in mailing genetic test kits (e.g., saliva kits mailed instead of buccal kits) that were later resolved. Participants also reported positive experiences talking with the genetic counselor, noting that the counselor was helpful, informative, and answered their questions. At KPMAS, participants reported finding value in the nurse coordinator calls, including the reminders, opportunities to have questions answered, and obtaining general information.

Participants reported several benefits of the Traceback program, noting that it prompted them to obtain genetic testing they may not have otherwise pursued, motivated them to pursue preventive care such as cancer screenings, and provided an opportunity to share the information with family members so they could learn more about their potential cancer risk. No participants reported any harms or negative consequences of their participation in the Traceback program.

Most participants, even those who did not undergo genetic testing themselves, expressed support for the idea of Traceback programs. Suggestions for improving the process included making the initial invitation more personalized to a patient’s history with cancer and prior genetic testing, providing more explicit guidance about the benefits and potential impacts of testing, better explaining costs and insurance coverage for genetic testing, and improving the readability and clarity of outreach and informational materials.

### 3.2. Implementer Perspective

We spoke with 17 implementers within the three clinical sites who were involved in administering the Traceback program. The roles of the implementers included nurses (*n* = 5), genetic counselors (*n* = 7), genetic counseling assistants (*n* = 4), and a medical geneticist (*n* = 1).

All implementers endorsed (via “Agree” response) the domains of both the acceptability (AIM) and appropriateness (IAM) measures. Overall, implementers were supportive of the Traceback program. They felt the program improved care quality by offering genetic testing to previously untested ovarian cancer survivors. Implementers felt that genetic testing offered valuable information to survivors and families that could improve cancer detection and management. Implementers attributed the lack of relatives completing cascade testing to the small numbers of tested patients with positive or pathogenic results. They felt that even receiving negative results was of value to participants and did not detract from the value of Traceback programs (Table 6).

Implementers reported that patients who completed Traceback testing were cancer survivors with biological relatives who could benefit from testing, survivors seeking to update their outdated genetic testing, and survivors who had previously refused testing but ultimately decided to undergo testing. They also reported that some patients expressed concern about the cost of both testing and follow-up visits, particularly for at-risk relatives.

Implementers felt that caring for Traceback program participants fit well into their regular clinical practice for genetic testing, follow-up, and recommendations for cascade testing in at-risk relatives. However, they noted several inefficiencies in the identification of Traceback-eligible individuals, such as the unexpected time and clinical expertise needed for manual chart review to confirm eligibility for Traceback testing.

Traceback program implementers noted several areas of focus for Traceback program improvement and sustainability. Firstly, more collaboration between clinical departments and physician education could help streamline the program and support a multidisciplinary approach. At KPMAS, where mailed genetic testing kits were used, implementers suggested that laboratory-based testing could reduce the amount of nudging needed to encourage kit return. Future directions for Traceback suggested by implementers included taking a public health approach, where program staff could handle eligibility assessment, outreach, and facilitation of testing. Such an approach could limit clinician time to testing and follow-up. Additionally, implementers suggested expanding the Traceback program to cover additional clinical conditions, increasing the potential clinical benefits. Several implementers mentioned the need for equitable access to Traceback programs for populations underrepresented in genomics research and clinical practice.

## 4. Discussion

We conducted a multisite pilot implementation study of Traceback genetic testing for individuals with ovarian, peritoneal, or fallopian cancer at three integrated health systems in the United States. We found that Traceback testing programs reached individuals with prior cancer diagnosis but no previous genetic testing (phase 1 of the Traceback framework) and were effective in identifying and communicating pathogenic variants to tested individuals (phase 2 of the Traceback framework). Programs were acceptable to both participants and implementers and thought to be of high clinical value. Implementation was feasible in this pilot study, but adequate amount of time and resources required to identify Traceback testing eligible individuals using administrative data and tumor registries are likely determinants of future sustainability. The pilot program did not result in cascade testing of at-risk relatives, despite including strategies of education about free cascade testing, reminder calls to probands (with seven pathogenic variants) at one site and offers to directly contact at-risk relatives at another site with two pathogenic variants. However, the small number of pathogenic variants identified limited the number of at-risk relatives eligible for cascade testing.

These findings contribute important early evidence about implementation of Traceback testing programs in U.S. settings. In the GRACE study, another Traceback program conducted in two U.S. health systems, participation was similar to our experience; in total, 26% of eligible living individuals completed testing, compared to 22% in our study. Using a custom gene panel, their rate of pathogenic variant detection was 21% of people completing testing, while ours was 8% [19]. Both studies found incidental findings from panel testing in genes with no evidence of association with ovarian cancer risk. Contributors to this lower yield may include unmeasured differences between acceptors and decliners of Traceback testing (e.g., family history of ovarian cancer, stage at diagnosis); differences in the testing panels; or ineligibility for Traceback testing because of high prior probability of positive result. Rates of VUS in our study (27%) were similar to those reported in other screening studies [20,21].

Traceback programs in other countries suggest additional strategies that could improve testing uptake. In a Traceback program conducted in Australia, in which archival tissue or stored blood from deceased probands with ovarian cancer was tested for pathogenic variants in 10 genes, 84/787 (11%) of samples were positive. When families were contacted directly with the result, 19/29 (66%) of relatives, who were not previously aware of the variant in the proband, accepted cascade testing [22]. In a Traceback study in Sweden, 302 women with breast cancer were identified using national health statistics and invited to Traceback testing, with 56% of those invited completing testing and a 9.7% detection rate of pathogenic variants based on testing for variants in eight genes [23]. Non-clinical workload (identifying and contacting probands, including reminders) was estimated at 4.6 min per invited woman and 8.2 min per woman who underwent genetic testing. Survey measures of acceptability were generally high.

We were surprised that no at-risk relatives received cascade testing using the free testing provided by the testing laboratory. It is possible that some at-risk relatives received testing through their own physician or other testing companies, or were not informed of the free testing by the Traceback participant. However, since no participants provided consent for us to contact at-risk relatives directly, we cannot evaluate the impact of that strategy. Cascade testing rates are generally low; a 2022 meta-analysis of at-risk relatives from over 87 studies found that only 41% of relatives completed cascade testing [24]. Direct contact of relatives is not a current standard of care at our clinical sites, which limits our ability to influence cascade testing behavior, particularly in at-risk relatives who receive care in other health systems. Although we used evidence-based strategies to improve reach to relatives, including offering direct contact of relatives at one site and reminder calls to probands to support their own sharing at another, we did not reach any relatives. It is possible that because of the length of time between diagnosis and Traceback testing, familial communication or cascade testing may have already occurred at the time of our outreach. Different, more intensive strategies may be warranted to directly reach at-risk relatives in the Traceback setting [25].

Our study provides important new data relevant to the implementation of Traceback programs. Phases 1 and 2 of our Traceback program (proband identification and testing of those not previously tested) reached more than half of eligible individuals, and was acceptable to people who were reached, whether or not they chose to be tested. Both participants and implementers were primarily driven by the potential clinical benefits to patients and families. It is worth noting that Traceback testing and follow-up did not appear to stress existing clinical workflows. However, identification of eligible individuals was unexpectedly time-consuming and would likely be a determinant of sustainability and scale-up to other cancers or health systems without adaptation. Having reminder calls was important to encouraging testing uptake and test completion for people who received mailed testing kits. Implementers universally saw the value in Traceback approaches for improving clinical outcomes and care quality and suggested applying Traceback methods to other tumor types for which genetic testing is indicated.

Strengths of our study include its multisite, real-world health system setting, the focus on recruiting diverse populations, the design of materials and outreach procedures based on patient preferences and local context at each site, and its focus on evaluating implementation outcomes, learning from both clinical stakeholders involved in implementation and individuals invited to Traceback testing. Our study team’s ancillary publications from this study also examined patient and family preferences for Traceback communications [12], legal aspects of using the U.S. public health exception for genetic testing programs like Traceback [26], and the cost-effectiveness of Traceback testing (in progress). The three health systems in this study represented a wide range of geographic, insurance coverage, and population demographics, increasing the potential generalizability of the results to other U.S. settings. However, results may not be generalizable to all health systems, such as safety-net health systems. Also, our study was limited to living cancer survivors, and so does not offer evidence on Traceback programs including relatives of deceased probands, such as consent and use of stored samples or testing of individuals who have a relative with ovarian cancer in the absence of test results in a proband [22,27]. Finally, the low prevalence of detected pathogenic variants in probands and different, context-specific strategies between sites limits our exploration of strategies for cascade testing in at-risk relatives, such as direct contact of relatives.

Future research could explore ways to directly engage at-risk relatives in the Traceback setting and more efficient ways to accurately identify Traceback-eligible individuals. Given the perceived value of Traceback programs, expanding the scope to more common cancers (e.g., breast or colorectal cancer) would allow observation of test uptake and identification of probands and cascade testing outcomes in larger sample sizes. This would also allow the study of the clinical and psychosocial outcomes of receiving Traceback results, including VUS, and of clinical intervention such as risk-reducing surgery in people with pathogenic variants identified though Traceback testing. Furthermore, Traceback approaches could be compared to population-based strategies such as population screening or population management approaches where untested probands and at-risk relatives continue to be offered testing at specific intervals.

## 5. Conclusions

This study demonstrates the feasibility, reach, acceptability, and potential effectiveness of Traceback testing for ovarian, fallopian, and peritoneal cancers. Further research is warranted on the sustainability of such programs, practices for facilitating cascade testing of at-risk relatives, and effectiveness of Traceback screening for other conditions.

## Figures and Tables

**Table 1:** Characteristics of Traceback program participants.

	Total, *n* (%)	KPWA *n* (%)	KPMAS *n* (%)	Geisinger *n* (%)	*p* Value, Difference Between Sites
Eligible	597	149	224	224	
Participated in testing	133	37	74	18	
Age at eligibility assessment, mean (SD)	69.8 (10.9)	73.8 (9.1)	68.0 (12.0)	69.8 (10.9)	0.029
Age at diagnosis, mean years (SD)	57.4 (10.3)	58.1 (10.5)	56.6 (9)	58.3 (11.8)	0.821
Years between eligibility assessment and age at diagnosis	13.2 (8.2)	13.2 (8.2)	13.2 (8.2)	13.2 (8.2)	13.2 (8.2)
Female	133 (100)	41(100)	74 (100)	18 (100)	NA
Race					<0.001
Asian	12 (9.0)	2 (4.9)	9 (12.2)	1 (5.6)	
Black	28 (21.1)	2 (4.9)	26 (35.1)	0 (0.0)	
Other race	7 (5.3)	2 (4.9)	5 (6.8)	0 (0.0)	
White	86 (64.7)	35 (85.4)	34 (45.9)	17 (94.4)	
Hispanic ethnicity	7 (5)	1 (2)	6 (8)	0	0.24
Ovarian cancer diagnosis	122 (92)	34 (83)	72 (99)	16 (89)	0.008
Peritoneal or fallopian cancer diagnosis	9 (8)	7 (17)	2 (1)	2 (11)	

**Table 2:** Results of Traceback genetic testing program.

	Total *n*	%	GE	%	KPWA	%	KPMAS	%
Potentially eligible individuals identified through administrative data, *n*	6386		1173		2274		2939	
Potentially eligible, alive and age 18+ at data pull, chart reviewed ^6^	1568		450		240		878	
Confirmed eligible for Traceback testing and attempted to reach, *n*	597	20% ^1^	224	50%	149	62%	224	10%
Eligible individuals reached, *n* ^7^	354	59% ^2^	129	58%	72	48%	153	68%
Interested in genetic counseling/testing, *n* (KPWA and Geisinger only)	74		33		41		NA	
Attended genetic counseling appointment	73		30		41		2	
Consented to genetic testing (no pretest counseling) (KPMAS only)	123		NA		NA		123	
Completed Traceback genetic testing, *n*	133	22% ^2^	18	8%	37	25%	74	33%
		38% ^3^		14%		51%		48%
Number of participants receiving positive result	10	2% ^2^	1	0.4%	2	1%	7	3%
		8% ^4^		5.6%		5%		9%
Variant of unknown significance (VUS) result	36	6% ^2^	5	2%	10	7%	21	9%
		28% ^4^		28%				28%
Total number of at-risk relatives identified	58		NA ^5^		21		37	
At-risk relatives completing cascade genetic testing, *n*	0				0		0	

GE = Geisinger; KPWA = Kaiser Permanente Washington; KPMAS = Kaiser Permanente Mid-Atlantic States. ^1^ Percentage potentially eligible. ^2^ Percentage of those confirmed eligible. ^3^ Percentage of those reached. ^4^ Percentage of those who completed Traceback testing. ^5^—not applicable; result not indicated for cascade testing. ^6^ —defined as currently receiving care (Geisinger) or current member (KPWA, KPMAS). ^7^—defined as study team spoke with person. KPMAS sent certified letters to all individuals attempted to reach.

**Table 3:** Genetic results, Traceback program (sites combined).

Genes with positive result ^1^ (Number of participants receiving result) (*n* = 10 participants)	Strong/definitive ovarian cancer association ^3^*:* *ATM (2), BRCA2 (2), RAD51D (2), BRCA1* No evidence of ovarian cancer association ^3^: *CHEK2 (3), TP53*
Variant of unknown significance (VUS) result genes ^2^ (*n* = 36 participants)	Strong/definitive ovarian cancer association ^3^: *ATM* ^4^ *(3), BRCA2 (3), BRIP1(2), PALB2, RAD51D, SMARCA4,* No evidence of ovarian cancer association ^3^: *NBN (3), POLE (3), BARD1(2), CDH1 (2), SDHA (2), APC, CHEK2, HOXB1, HOXB13, KIF1B, KIT, MSH3, NF1, NTHL1, PDGFRA, POLE/HOXB1, RAD50, RET, SDHC, TSC2*

^1^ One participant received positive variants in two genes. ^2^ Three participants received multiple VUS. ^3^ Per Clinical Genome Resource or National Comprehensive Cancer Network as of January 2025. ^4^ One participant had an *ATM* VUS and *APC* VUS.

**Table 4:** Demographics of interview participants (*n* = 47).

	N	% or Range
Total interviewees	47	100%
Geisinger	18	38.3%
KPMAS	13	27.7%
KPWA	16	34.0%
Median age	69	(48–90)
Female	47	100%
Race/ethnicity		
Asian	4	8.5%
Black	6	12.8%
White	36	76.6%
Hispanic ethnicity	0	0.0%
4-year college graduate or more	27	57.4%
Annual income $50K or less	11	23.4%
Currently working for pay	8	17.0%
Currently married	26	55.3%
Completed Traceback genetic testing	30	63.8%

Abbreviations: KPMAS = Kaiser Permanente Mid-Atlantic States; KPWA = Kaiser Permanente Washington.

**Table 5:** Patient experiences with and reflections on Traceback program.

Category	Them	Example Quotes
Reaction to initial outreach	Positive reaction	My reaction is really positive. Because after like you looking out for me, you know, when you invite me to do genetic testing you’re not only looking out for me, you’re looking out for my family. So I feel good, you know, taking genetic testing is not only for me. KPWA00032 (Acceptor)
		I didn’t have any qualms about it. I was happy that it was my… you know my Kaiser doctor. I trust them. I don’t know how I would’ve felt had I gotten it from some other program. I don’t know, but it was all through Kaiser so I didn’t have any… any, any hesitation about it. KPMAS0008 (Acceptor)
	Negative reaction	First off I had ovarian cancer in 1973. I’ve been with multiple entities of what is now Kaiser forever and you’re just now asking me this?… If you’re going to tell me what you’re looking for, if you’re going to tell me how this is going to benefit and don’t tell me it’s free. Because Kaiser doesn’t do nothing for free… To say it’s free you’re getting my DNA which is mine and ask me to give it to you for free, so that’s about the only free there is. KPMAS0001 (Decliner)
		Well, what was going through my head was if you want me to come down there and get another needle you are nuts, that was going through my head before she even said anything. GE90692 (Decliner)
	Surprised by outreach	I was somewhat surprised, and I guess I felt as though oh what is it about me that makes them want me to do this? GE00345 (Decliner)
	Did not remember	I’m sorry, but I don’t know if my husband threw that away. I don’t remember seeing a letter if he answered the phone. GE00185 (Decliner)
Genetic testing and counseling experience	Easy, straightforward testing process	It was pretty straightforward. I talked with the nurse and that was simple. The testing kit came as I was told it would and did the test. In an era of COVID testing this just seemed like one more set of swabs and containers. So it didn’t really seem very exotic. KPMAS0002 (Acceptor)
	Informative conversations with genetic testing staff	I think it was great. Everybody was really nice and really informative and answered all the questions. And explained what was going in really clear terms so that I completely understood it. KPWA00059 (Acceptor)
Benefits of program	Prompt to get genetic testing that otherwise would not have pursued	I received the letter and I dismissed it at first because I thought I don’t feel I need to do it. …Then one day I thought you know I have a child, I have a daughter. And I thought perhaps I should do this and gather more information for her. KPMAS0005 (Acceptor)
	Provided relevant risk information to family members	I encouraged them both to get tested. You know, and like I say, one daughter did, she did not have that genetic mutation. And hopefully the other one doesn’t have it either, but she has not completed the genetic testing. KPWA00131 (Acceptor)
	Motivation to pursue preventive health actions	I’m going to get yearly mammograms and yearly breast exams. And just, you know, do what we can to catch it early if it’s going to happen. Hopefully it won’t. KPWA00059 (Acceptor)
Suggestions for improvement	Personalize outreach to proband’s health history	So I think the only confusion that I had was that I already had the genetic testing, so I wasn’t quite sure why they were reaching out to me again. KPWA00208 (Acceptor)
		I don’t recall that there was any… any indication of awareness of how long in the past my cancer diagnosis was which you know it’s kind of historical information. KPMAS0002 (Acceptor).
		The only question I have is what took them so long to decide they needed to do a genetic testing… Because a lot of people have died from ovarian cancer in 20 some years. KPWA00089 (Decliner)
	Explicitly explain benefits and potential impacts of testing	I would have appreciated maybe some more up-front information about how this can affect peoples’ lives if they discover things in their genes that are--could be passed on. So, you know, I think just a little more education with why you’re--why we need to do this. KPWA00037 (Acceptor)
		I think they could have identified, you know, these are the things that we might find out that would be helpful. You know, whether you could pass this along, whether somebody else in your family or immediate family might already have this, or they should get a particular test, or things like that. I have a couple of nieces out there, grandnieces, but because they’re not direct genetically, I just assumed that that wouldn’t affect them. So, if there is something that could be useful to them, then that would be good for me to pass along. KPWA00035 (Decliner)
		Well, people always want to contribute, you know. If somehow taking my blood would be greatly helpful in say prevention or detection, then that’s fine. So I’d have to be convinced that it’s worth my time, worth my effort. KPWA00066 (Decliner)
	Provide more information about cost and coverage of testing	I had no idea what it would cost, so I couldn’t decide whether I wanted to go ahead and do it anyway because nobody could help me and tell me what out of pocket costs I would be looking at. So, therefore, I did not do the genetic testing. GE00345 (Decliner)
	Improve readability and clarity	Well, the letters were sort of wordy and vague. I mean, I understood what was going on, but I could see how they could confuse people who are less scientifically literate. KPWA00070 (Acceptor)
	Offer ways to address logistical barriers	I did read through it, and I found it a little difficult on how I could possibly participate. I have no means of getting, you know, to where I can do bloodwork or anything. KPWA00089 (Decliner)

Abbreviations: GE = Geisinger; KPMAS = Kaiser Permanente Mid-Atlantic States; KPWA = Kaiser Permanente Washington.

**Table 6:** Implementer perspectives on Traceback program.

Theme	Exemplar Quotes
Perceptions of the traceback program	I think the benefits of a program like this, something that comes to mind is we often, in my opinion, miss this specific patient population group for many reasons. They either are diagnosed a bit later in life, and they pass away fairly after their diagnosis or even if they do seek treatment, sometimes they are way too overwhelmed and busy to fit genetics in or maybe their physicians are not recommending genetics for whatever reason, so I think there’s many reasons why they don’t come to see us. So, I think a program like this pulls in this specific patient group where genetic testing is really beneficial for them. Genetic Counselor, Geisinger I think if we find better paths to go back and revisit some of these patients that have had really difficult diseases, that’s part of our mission. Geneticist, KPWA
Traceback program value	I think a lot of people who are diagnosed with cancer, they don’t realize that this [genetic testing] is an option for them, especially because they meet guidelines and that this could be a really great thing for their family and for kind of getting an answer. Maybe for future screening and future preventative care as well. So I think knowledge is power. So, you know, even if it’s late knowledge in there, in some people’s minds, it might be kind of late finding out. But I think any knowledge at any point is great…So I just think it’s a great thing overall if we can explain it in a way that suits them and let them kind of decide what they want to do. Genetic Counseling Assistant, Geisinger I think the cascade testing which we talked about was the biggest benefit…I think that’s why most people wanted to do testing and there was a huge benefit. Genetic Counselor, KPWA
Acceptability of outreach timing	…When someone is diagnosed with cancer and facing a cancer diagnosis, there’s so many people contacting them, there are so many things they have to do, and then this was great because things had died down, it was much later, and they could focus on it, and possibly hear it more, so I really like this approach…it could really help. There may be people who decline, that’s fine, but I think it could really help. Genetic Counselor, Geisinger
Perceptions of patient motivation to test	I ended up seeing a lot of people who had had an outdated genetic test, like the BRCA only or maybe a smaller panel than what we now have. I think though, overall, the most common reason that people decided to go forward with testing is to if they had children or other at-risk relatives, that was their strongest motivation. Genetic Counselor, KPWA Both of the patients I saw were elderly…so their ovarian cancer diagnoses were between 25 and 30 years prior, when genetic testing wasn’t necessarily on the market, as widespread, their oncology team at the time might not have known that’s something that can be done, what insurance and cost look like…But, I think for both of them, their main benefit of testing for them was their children and their family. Both had children, males and females, they were wanting [a] great opportunity to impact their care, both recognizing it probably didn’t mean too much for them at their current age and health status. Genetic Counselor, Geisinger
Traceback workflow feedback	I didn’t really notice any differences in the patients at all or the work I was conducting. It all seemed pretty similar to other patients I would see through the cancer clinic. Genetic Counseling Assistant, Geisinger I would say that the only thing that would make this more perfect would be to have them come in, get a cheek swab in the office and leave so they don’t have to deal with sending the specimen back. Nurse Coordinator, KPMAS
Traceback program sustainability	I think working side by side with cancer genetics—that already has a really good process of getting these patients seen—it is perfect. I mean, this is just another way to capture a group of people that might be missed or might not know that they have this option. Genetic Counseling Assistant, Geisinger …one of the things that I learned from Traceback is that it is essential that we have training for all providers who interact with patients with this type of cancer or any type of serious cancer that we, give them the correct information. And if a provider can’t do that directly, is to know that you collaborate with other providers to do that work. Geneticist, KPWA
Cost of testing	I got a lot of positive feedback from patients about how happy and excited they were that this was offered to them free of charge. Nurse Coordinator, KPMAS There seemed to be a good amount of times when they expected that this should be covered because I was contacted and it should be free, so that was the big difference I saw. Genetic Counselor, Geisinger
Future directions	I mean, even if there were something like population screening, I think it would still be valid to go back and recontact individuals and check on how they’re doing. Genetic Counselor, KPWA I would be interested in maybe researching more into how we can make the genetic counseling model a little bit more inclusive to other groups. Genetic Counseling Assistant, KPWA

## Data Availability

The raw data supporting the conclusions of this article will be made available by the authors on request.

## Supplementary Materials

The following supporting information can be downloaded at: https://www.mdpi.com/article/10.3390/cancers17071154/s1, Semi-structured interview guide.
