# Supplementary material for: Implementation of a Traceback Testing Program for Ovarian Cancer: Findings from the FACTS Study
Source: Cancers (Basel). 2025 Mar 29;17(7):1154. doi: 10.3390/cancers17071154 (PMC11988076; doi:10.3390/cancers17071154)
Supplement: Supplementary file 1 [file cancers-17-01154-s001.zip › cancers-3477091-supplementary.pdf]

__**Interview Guide for Proband Participants in Traceback Program**__
*Note: each site (Geisinger, KP Washington, KP Mid-Atlantic States) made slight modifications to this interview protocol to align with their site's context and processes.*

1. **Can you tell me why you decided to participate in this study?**

2. **Could you tell me about your experience with ovarian** [or fallopian or peritoneal] **cancer?**
   *[optional prompts: when did you have it? How did it go? How are you doing now?]*

3. **Now I'll ask you some questions about some information you received about genetic testing. You may have received an invitation to get genetic testing from a letter, a patient portal message, a phone call, or you may have seen a poster in a clinic. The information might have used the term "Traceback program." Do you remember receiving this invitation?**

4. **What was going on in your life when the Traceback program contacted you about getting genetic testing?**

5. **Walk me through your experience of being contacted about getting genetic testing**
   *[optional prompts: Who first contacted you? How did they contact you (e.g., letter, phone call, etc.? When did this happen? What were you doing?]*

6. **What was your initial reaction to the [letter / phone call / message] inviting you to get genetic testing? Did your reaction change over time? If so, how?**
   *[optional prompts: Was your reaction positive, negative, neutral? What did you do after receiving the letter / phone call / message?]*

7. **What questions did you have at that time? Did you get the answers you needed? How?**
   *[optional prompts: Did you speak with a genetic counselor or someone else (like a nurse)/go online/talk to a relative or friend about whether or not you should get testing? How did you feel about speaking with the genetic counselor versus another staff member before and after testing?]*

8. **Tell me about how you decided whether or not to get genetic testing.**
   *[optional prompts: Why did you decide to get / not get genetic testing? What did you consider when you were making your decision? Did you talk with someone about it?]*

9. **[Acceptors only] What was the genetic testing process like for you?**
   *[optional prompts: How did you feel about the process? Was it easy to do / hard to do? What parts were easy or hard?]*

10. **[Acceptors only] What happened after you received your genetic test results?**
    *[optional prompts: What did you do? Who did you talk to? Did you talk with your family members? Did you go online? Look anything up? Did your family members do anything with this information?]*

11. **[Acceptors only] Did you speak with any of your relatives about genetic testing?**
    a. **[If yes] Which relatives? How did those conversations go? Do you know if any of your relatives are planning to get genetic testing?**
    b. **[if no] are you comfortable sharing the reasons why not?**

12. **[Acceptors only] How do you feel about the cost of testing? Was there anything surprising or unexpected about the cost of testing?**
    *[optional prompts: Was it more / less expensive than you thought? Did you understand what the costs would be in advance?]*

13. **[Acceptors only] How are you feeling now about your genetic test results?**
    *[optional prompts: If given the chance to repeat the process, would you make the same choice to get testing? Is there anything you would change about your experience with testing? Did your results influence your feelings about participating in this study?]*

14. **What would you suggest changing or improving about [the invitation to get genetic testing / the process / the Traceback program]?**

15. **Overall, how do you feel about your experience with [being invited to get genetic testing / the Traceback program]?**
    a. **How useful do you think this program is?**
    b. **Do you think it would be useful for other people?**

16. **What else is important for me to know?**
